# Molecular Characteristics of *Streptococcus pyogenes* Isolated From Chinese Children With Different Diseases

**DOI:** 10.3389/fmicb.2021.722225

**Published:** 2021-12-09

**Authors:** Dingle Yu, Yunmei Liang, Qinghua Lu, Qing Meng, Wenjian Wang, Lu Huang, Yanmin Bao, Ruizhen Zhao, Yunsheng Chen, Yuejie Zheng, Yonghong Yang

**Affiliations:** ^1^Microbiology Laboratory, National Center for Children’s Health, Beijing Pediatric Research Institute, Beijing Children’s Hospital, Capital Medical University, Beijing, China; ^2^Shenzhen Children’s Hospital, Shenzhen, China; ^3^Beijing Chaoyang Hospital Affiliated to the Capital Medical University, Beijing, China

**Keywords:** *Streptococcus pyogenes*, disease, China, child, superantigen, antibiotic resistance, *emm* type

## Abstract

*Streptococcus pyogenes* is a bacterial pathogen that causes a wide spectrum of clinical diseases exclusively in humans. The distribution of *emm* type, antibiotic resistance and virulence gene expression for *S. pyogenes* varies temporally and geographically, resulting in distinct disease spectra. In this study, we analyzed antibiotic resistance and resistance gene expression patterns among *S. pyogenes* isolates from pediatric patients in China and investigated the relationship between virulence gene expression, *emm* type, and disease categories. Forty-two representative *emm*1.0 and *emm*12.0 strains (*n* = 20 and *n* = 22, respectively) isolated from patients with scarlet fever or obstructive sleep apnea-hypopnea syndrome were subjected to whole-genome sequencing and phylogenetic analysis. These strains were further analyzed for susceptibility to vancomycin. We found a high rate and degree of resistance to macrolides and tetracycline in these strains, which mainly expressed *ermB* and *tetM*. The disease category correlated with *emm* type but not superantigens. The distribution of *vanuG* and virulence genes were associated with *emm* type. Previously reported important prophages, such as φHKU16.vir, φHKU488.vir, Φ5005.1, Φ5005.2, and Φ5005.3 encoding streptococcal toxin, and integrative conjugative elements (ICEs) such as ICE-emm12 and ICE-HKU397 encoding macrolide and tetracycline resistance were found present amongst *emm*1 or *emm*12 clones from Shenzhen, China.

## Introduction

*Streptococcus pyogenes* (group A *Streptococcus*, GAS) is an important gram-positive bacteria that ranks among the 10 main causes of death from infectious diseases worldwide, with more than 517,000 deaths annually (Carapetis et al., 2005). GAS causes a wide spectrum of clinical diseases ranging from mild pharyngitis to life-threatening invasive infections (Carapetis et al., 2005; Walker et al., 2014). Although antibiotics are effective and widely used for treating GAS infections, antibiotic resistance, especially to macrolides, is increasing in several countries (Lu et al., 2017; Bhardwaj et al., 2018). The rise of antibiotic resistance leads to an increase in mortality, which has become a public health issue of global concern (Wajima et al., 2014; Silva-Costa et al., 2015). This issue has also received close attention in China (You et al., 2018, 2020). Genotyping is an effective method for monitoring bacterial strains in microbiology research (Steer et al., 2009), and sequence analysis of the *emm* gene is currently used for GAS genotyping (Facklam et al., 1999; Gherardi et al., 2018). In recent years, *emm* cluster analysis has also been widely used in GAS molecular epidemiology analysis. The type-specific M protein, encoded by the *emm* gene, and superantigens (SAgs), encoded by the *sAg* genes, are important virulence factors in *S. pyogenes* (Golińska et al., 2016). Currently, stains of *S. pyogenes* are often tested for the expression of *speA*, *speC*, *speG*, *speH*, *speI*, *speJ*, *speK*, *speL*, *speM*, *ssa, smez*, and the enzyme-encoding *speB* and *speF* genes as genes encoding SAgs, even though *speB* and *speF* have been confirmed to encode cysteine protease and streptococcal DNase proteins (Strus et al., 2017). However, the correlations between *sAg* distribution, *emm* type, and disease spectrum for GAS have not yet been established (Rantala et al., 2012; Imöhl et al., 2017; González-Abad and Alonso Sanz, 2020). In this study, the antimicrobial sensitivity of GAS strains as well as the relationships among *sAg* distribution, *emm* types, and disease categories were analyzed. Moreover, 42 representative strains of the two main epidemic *emm* types were analyzed for population structure, genetic diversity, phylogeny, and susceptibility to vancomycin.

## Materials and Methods

### Bacterial Strains and Antimicrobial Susceptibility Testing

A total of 342 GAS strains were isolated from children aged < 18 years who were admitted to Shenzhen Children’s Hospital from 2016 to 2018 for treatment of one of 15 diseases. Of these strains, 87 were isolated in 2016 (4 invasive and 83 non-invasive strains), 138 were isolated in 2017 (13 invasive and 125 non-invasive strains), and 117 were isolated in 2018 (15 invasive and 102 non-invasive strains). The strains were isolated from 262 throat swabs, 47 sputum samples, 21 pus samples (abscess, surgical wound infections, and skin burn infections), 5 wound secretions, 3 vulvar secretions, 3 blood samples, and 1 urine sample. The *streptococcus* grouping kit (Oxoid Limited) was used to identify these strains, as previously reported (Liang et al., 2012).

We tested the susceptibility of the 342 strains to antimicrobial agents including penicillin, azithromycin, erythromycin, clarithromycin, clindamycin, tetracycline, levofloxacin, and chloramphenicol (Oxoid Limited). Susceptibility to vancomycin was analyzed in 42 representative strains. Minimum inhibitory concentration (MIC) values were determined according to the guidelines of the Clinical and Laboratory Standards Institute (CLSI) (2019) by using the broth dilution method. Quality control was performed using *Streptococcus pneumoniae* ATCC 49619, which was provided by the Clinical Test Center of the Ministry of Health and maintained by the Microbiology Laboratory of Shenzhen Children’s Hospital.

### DNA Extraction and Detection of Superantigens and Macrolide and Tetracycline Resistance Genes

Genomic DNA was obtained from freshly grown GAS using a Chelex-based DNA extracting kit for genetic analysis. Thirteen SAg-coding genes (*speA*, *speB*, *speC*, *SpeF*, *speG*, *speH*, *speI*, *speJ*, *speK*, *speL*, *speM*, *ssa*, and *smeZ*), three genes encoding resistance to macrolides (*ermB, ermA*, and *mefA*), and tetracycline (*tetM*) were detected by polymerase chain reaction (PCR) using a previously reported protocol (Chatellier et al., 2000; Igwe et al., 2003; Rivera et al., 2006; Lintges et al., 2007; Pérez-Trallero et al., 2007). The DNA extraction kit, PCR reagents, and primers were all obtained from Shanghai SBS Genetech Co., Ltd. (China).

### *Emm* Sequence Typing

The *emm* sequence types were determined using a previously reported protocol^1^. Amplicons were sequenced by Guangzhou BGI Genomics Co. Ltd. *Emm* type was determined based on the sequence identity (> 95%) of the first 180 bp of the *emm* gene between the tested sequence and the reference *emm* gene.

### Whole-Genome Sequencing and Phylogenetic Analysis

We selected 42 strains that represented the major *emm* types for WGS according to the following criteria: (i) the major *emm* types (*emm*1.0, *emm*12.0), (ii) the two most common diseases [scarlet fever and obstructive sleep apnea syndrome (OSAS)], and (iii) 3 strains each year from 2016 to 2018. For each subtype of *emm*1 and *emm*12, we choose one strain from each year, and then chose strains from different seasons in each year. Forty-two strains isolated from children with OSAS or scarlet fever (20 *emm*1.0 strains and 22 *emm*12.0 strains) were used for the WGS analysis. Genomic DNA was sequenced using the DNBSEQ platform (BGI-Shenzhen, China). DNA quality was assessed by electrophoresis, and then the DNA was fragmented and processed by end repair, A-tailing, adapter ligation, DNA size selection, circulation, and DNA nanoball formation according to an in-house library. DNA libraries with an insert size of 300 bp were sequenced using paired-end 100-bp reads (PE100). Low-quality sequences were trimmed using SOAPnuke (Chen et al., 2018). The remaining short reads were assembled into contigs using SPAdes version 3.11.1 (Lagesen et al., 2007).

Genes were predicted using Glimmer 3.02 (Delcher et al., 2007). Virulence genes were identified by searching against the Virulence Factor Database (Liu et al., 2019), Antibiotic Resistance Genes Database (Liu and Pop, 2009), and Comprehensive Antibiotic Resistance Database (Alcock et al., 2020). The core genes of the sequenced and reference genomes were identified by clustering proteins with a sequence identity > 50% and a coverage > 70% using CD-HIT (Fu et al., 2012). Single nucleotide polymorphisms (SNPs) among the core genes were aligned pairwise and subjected to phylogenetic tree inference using TreeBeST and the NJ method (TreeSoft, 2021).

### Statement of Ethics

This study was approved by the research ethics committee of the Shenzhen Children’s Hospital. Informed consent was obtained from patients or their guardians before sample collection.

### Statistical Analysis

Data were analyzed using SPSS version 22.0. Differences in the distributions of diseases and *emm* types and comparisons between diseases and a specific *emm* type were analyzed using the independent-samples Kruskal-Wallis test. The exact Mann-Whitney *U*-test was applied to identify differences in distributions between streptococcal *sAg* expression, *emm* types, and disease categories. Two-sided *P-*values < 0.05 indicated a statistically significant difference between groups. Values of *P*_adj_ < 0.05/m indicated that a within group difference was statistically significant, where m represents the total number of Bonferroni corrections within the group.

## Results

### Antimicrobial Susceptibility Patterns and Resistance Genes

All strains were highly susceptible to penicillin and chloramphenicol, whereas 98.5% of the tested strains were sensitive to levofloxacin. The results regarding the rates and degree of resistance to azithromycin, erythromycin, clarithromycin, clindamycin, and tetracycline are shown in Table 1. In addition, 90.9 and 6.1% of strains expressed *ermB* and *ermA*, respectively. Additionally, 16.4% were *mefA*-positive, and 85.4% were *tetM*-positive. All 42 representative strains were susceptible to vancomycin.

**TABLE 1:** Susceptibility of *S. pyogenes* strains (*N* = 342) to antimicrobial agents.

Antibiotic	R% (N)	I% (N)	S% (N)	(μg/mL)			
	
				Breakpoint	MIC50	MIC90	MIC range
Penicillin	0	0	100 (342)	S ≤ 0.125	0.004	0.008	0.001–0.03
Azithromycin	91.5 (313)	0	8.5 (29)	S ≤ 0.5 R ≥ 2	>256	>256	0.015–512
Erythromycin	91.2 (312)	0	8.8 (30)	S ≤ 0.25 R ≥ 1	>256	>256	0.0625–512
Clarithromycin	90.6 (310)	0.9 (3)	8.5 (29)	S ≤ 0.25 R ≥ 1	>256	>256	0.0375–512
Clindamycin	90.6 (310)	0	9.4 (32)	S ≤ 0.25 R ≥ 1	128	256	0.015–512
Tetracycline	86.5 (296)	0	13.5 (46)	S ≤ 2 R ≥ 8	64	64	0.125–64
Levofloxacin	0	1.5 (5)	98.5 (337)	S ≤ 2 R ≥ 8	1	2	0.25–4
Chloramphenicol	0	0	100 (342)	S ≤ 4 R ≥ 16	2	4	1–4

*S, Susceptible; I, Intermediate; R, Resistant.*

### Distribution of *emm* Genotypes and Their Correlation With Disease Categories

**FIGURE 1:**
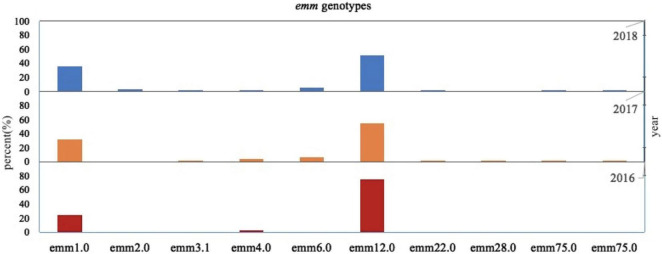
Distribution of *Streptococcus pyogenes emm* genotypes in isolates collected from pediatric patients in China from 2016 to 2018.

**FIGURE 2:**
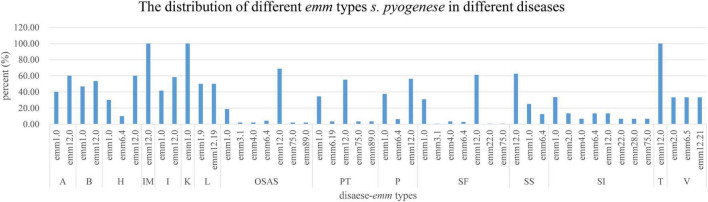
Distribution of *Streptococcus pyogenes emm* types in isolates from children with different diseases.

In this survey, 10 *emm* types, including 7 subtypes, were identified among the 342 GAS strains from 15 different diseases. Figures 1, 2 show the details of the 10 *emm* types, as well as the 7 subtypes, for the different disease categories in strains collected between 2016 and 2018. The distribution of disease categories differs significantly between *emm*12.0 and *emm*2.0 stains (Table 2).

**TABLE 2:** Relationship between *emm* types of *S. pyogenes* and disease categories.

Disease	No. of cases	*emm*1.0	*emm*12.0	*emm2.0*	*emm*3.1	*emm*4.0	*emm*6.0	*emm*22.0	*emm*28.0	*emm*75.0	*emm*89.0
Arthritis	5	2	3	0◎	0	0	0	0	0	0	0
Bronchitis	15	7	8	0*※	0	0	0	0	0	0	0
HSP	10	3	6	0^￥*^	0	0	1	0	0	0	0
Impetigo	12	5	7	0^¤^°	0	0	0	0	0	0	0
OSAS	48	9	33◎	0^©^ᵿ	1	1	2	0	0	1	1
Pharyngeal tonsillitis	29	10	16*	0 ^® ø^	0	0	1	0	0	1	1
Pneumonia	16	6	9	0^◊¢^	0	0	1	0	0	0	0
Scarlet fever	175	54	107※	0	1	6	5	1	0	1	0
Sepsis	8	2	5	0	0	0	1	0	0	0	0
Soft tissue infection	15	5	2^◎＊※^	2^＊*°¢øᵿ^	0	1	2	1	1	1	0
Vulvitis	3	0	1	1^◎※＄￥§¤©®◊^	0	0	1	0	0	0	0
IM	2	0	2	0^§^	0	0	0	0	0	0	0
Lymphadenitis	2	1	1	0^$^	0	0	0	0	0	0	0
Tympanitis	1	0	1	0	0	0	0	0	0	0	0
Kawasaki disease	1	1	0	0	0	0	0	0	0	0	0
Independent-samples	*P*-value	0.615	<0.001	<0.001	1.000	0.858	0.438	0.925	0.083	0.986	0.915
Kruskal-Wallis test	*P*_adj_ value	NA	<0.05/105	<0.05/105	NA	NA	NA	NA	NA	NA	NA

Data represent number of cases. OSAS, obstructive sleep apnea hypopnea syndrome; HSP, Henoch–Schöenlein Purpura; IM, infectious mononucleosis. ◎, ＊, ※, ＄, ￥, §, Ó, Ò, ◊, *, °, ¤, ¢, ø, and ᵿ, and ᵿ indicate that the distribution of disease categories for specific emm types was significantly different after Bonferroni correction (P_adj_ value < 0.05/105).

### Correlations Between Superantigen Expression Profiles, Disease Categories, and *emm* Types

The positivity rates for *speA, speB, speC, speF, speG, speH, speI, speJ, speK, speL, speM, ssa*, and *smeZ* differed among the strains, and the distributions of *speA*, *speH*, *speI*, and *speJ* expression were related to the *emm* types (Table 3). The distributions of *sAgs* in different disease categories did not differ significantly (Table 4).

**TABLE 3:** Distribution of *sAgs* in *S. pyogenes* strains according to *emm* types.

*emm* types	No. of strains	*speA*	*speB*	*speC*	*speF*	*speG*	*speH*	*speI*	*speJ*	*speK*	*speL*	*speM*	*ssa*	*smeZ*
*emm*1.0	105	93^◎＊※＄*^	104	104	102	94	4◎ *	4◎ * ※	98^◎＊※＄*°¤oe^	2	1	2	104	104
*emm*2.0	3	0◎	3	3	3	3	1	1	0◎	2	2	2	1	1
*emm*3.1	2	2^Δ^	2	0	2	0	0※	0	0*	2	0	2	0	2
*emm*4.0	8	2*^œ^	8	8	7	2	0^$^	0^$^	0※	2	4	2	6	7
*emm*6.0	14	12^2￥^	14	14	14	9	1*	1^￥^	1^＄^	12	2	12	4	14
*emm*12.0	201	5^※Δ￥°oe^	199	199	198	176	171^◎※＄*°oe^	169^◎＄￥Δ^	6^⁎^	3	7	2	198	199
*emm*22.0	2	1°	2	2	2	2	0°	0	0°	0	0	0	2	2
*emm*28.0	1	0	1	1	1	1	0	1*^Δ^	1	0	0	0	0	1
*emm*75.0	4	1^$^	4	3	4	4	2*	3※	0^œ^	3	2	3	1	4
*emm*89.0	2	0*	2	1	2	2	0^œ^	0	0^¤^	0	0	0	1	2
n (%)	N = 342	33.9	99.1	98.0	98.0	85.7	52.3	52.3	31.0	7.6	5.3	7.3	92.4	98.2
Mann-Whitney U test	U	1935.00	504.000	955.000	944.500	6715.50	4322.50	4114.00	1382.50	3637.50	2719.00	3430.50	3888.00	826.50
	Z	−14.728	−0.030	−0.959	−1.005	−0.823	−12.828	−13.088	−15.013	−1.108	−0.551	−1.276	−0.179	−0.863
	*P*	<0.001	0.976	0.338	0.315	0.411	<0.001	<0.001	<0.001	0.268	0.582	0.202	0.858	0.388

◎, ＊, ※, ＄, ￥, *, D, oe, °, and ¤ indicate a statistically significant difference after Bonferroni correction (P_adj_ < P/45).

**TABLE 4:** Distribution of *sAgs* in *S. pyogenes* strains isolated from children with different diseases.

Disease	No. of cases	*speA*	*speB*	*speC*	*speF*	*speG*	*speH*	*speI*	*speJ*	*speK*	*speL*	*speM*	*ssa*	*smeZ*
Arthritis	5	2	5	5	5	5	3	3	2	0	0	0	5	5
Bronchitis	15	8	15	15	15	15	6	6	7	2	3	2	13	14
Henoch–Schöenlein purpura	10	4	10	9	9	7	4	4	3	1	0	1	9	10
Impetigo	12	5	11	12	12	9	6	6	5	0	0	0	12	12
OSAS	48	11	48	45	48	41	30	29	9	4	6	4	43	48
Pharyngeal tonsillitis	29	11	29	29	27	26	15	15	12	2	1	2	26	29
Pneumonia	16	6	16	16	16	12	8	9	5	1	0	1	15	16
Scarlet fever	175	54	174	173	172	148	95	94	52	8	5	8	168	172
Sepsis	8	3	8	8	8	7	5	5	2	1	1	1	8	8
Soft tissue infection	15	9	15	14	15	14	2	3	7	5	2	5	9	13
Vulvitis	3	1	3	3	3	3	2	2	0	1	0	1	3	3
Infectious mononucleosis	2	0	2	2	2	2	2	2	0	0	0	0	2	2
Lymphadenitis	2	1	2	2	2	2	0	0	1	1	0	0	2	2
Tympanitis	1	0	0	1	1	1	1	1	0	0	0	0	1	1
Kawasaki disease	1	1	1	1	0	1	0	0	1	0	0	0	1	1
Mann-Whitney *U*-test	U	13020.0	422.5	969.0	676.5	6913.0	13983.5	14147.5	12369.5	3509.0	2453.5	3531.5	3906.5	703.0
	Z	−0.109	−0.543	−0.846	−0.817	−0.446	−0.713	−0.520	−0.176	−1.331	−1.220	−0.975	−0.127	−1.368
	*P*	0.913	0.596	0.414	0.423	0.657	0.476	0.603	0.860	0.184	0.224	0.332	0.900	0.186

*OSAS, obstructive sleep apnea hypopnea syndrome.*

**FIGURE 3:**
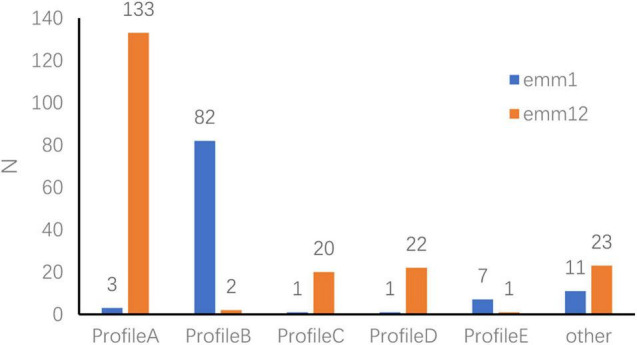
Superantigen-encoding gene expression profiles in *emm*1.0 and *emm*12.0 types. Profile A: *speA–, speB*+, *speC*+, *speF*+, *speG*+, *speH*+, *speI*+, *speJ–, speL–, speM–, ssa*+, *smeZ*+, *speK–.* Profile B: *speA*+, *speB*+, *speC*+, *speF*+, *speG*+, *speH–, speI–, speJ*+, *speL–, speM–, ssa*+, *smeZ*+, *speK–.* Profile C: *speA–, speB*+, *speC*+, *speF*+, *speG*+, *speH–, speI–, speJ–, speL–, speM–, ssa*+, *smeZ*+, *speK–.* Profile D: *speA–, speB*+, *speC*+, *speF*+, *speG–, speH*+, *speI*+, *speJ–, speL–, speM–, ssa*+, *smeZ*+, *speK–.* Profile E: *speA*+, *speB*+, *speC*+, *speF*+, *speG–, speH–, speI–, speJ*+, *speL–, speM–, ssa*+, *smeZ*+, *speK–.*

Among the 342 strains, 79.5% expressed six or more *sAgs*. The five major gene profiles (A–E) associated with *emm* types were identified according to the *sAg* combinations (Figure 3).

### Genetic Diversity in Representative *Emm*1.0 and *Emm*12.0 Clones

**FIGURE 4:**
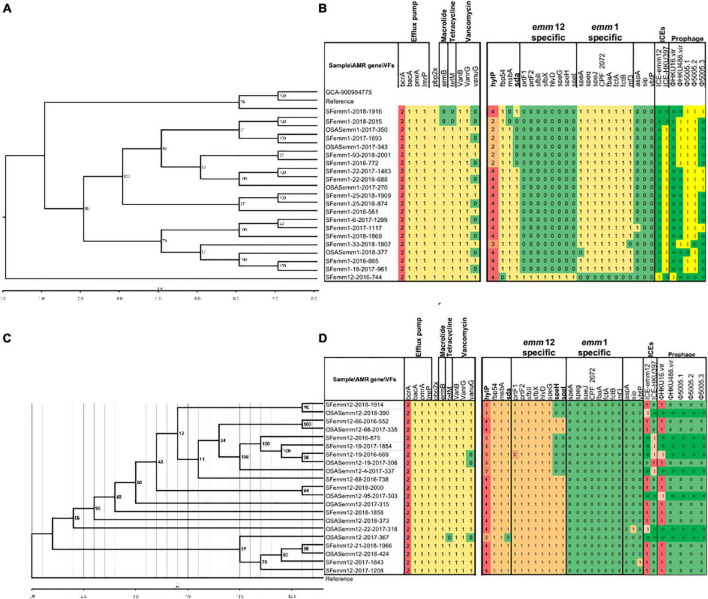
Phylogenetic relationship of selective *emm*1 and *emm*12 strains using whole genome core-gene SNPs **(A,C)** and their antimicrobial drug resistance gene and virulence factor variation profiles (b,d). References indicate *S. pyogenes* strain MGAS5005 (Genbank accession CP000017.2) and *S. pyogenes* strain TJ11-001 (CP028148.1), respectively **(B,C).** The *S. pyogenes* strain Bra048, assembly accession GCA_900984775, is also included in the phylogeny of *emm*1 for reference. Strains containing 2 copies (*emm*1) and 3 copies (*emm*12), rather than the majority 4 copies, formed clusters and are indicated in bold font. The numbers in cells **(B,D)** indicate gene copy numbers.

The analysis of antimicrobial resistance gene expression in the genome sequences identified 10 genes (*ermB*, *tetM*, *pbp2x*, *bcrA, bacA, pmrA*, *lmrP, vanB, vanrG*, and *vanuG*), which included two copies of *bcrA* and a single copy of each of the other nine genes. The frequency of *vanuG* expression was significantly higher in *emm*12.0 strains (18/22) than in *emm*1.0 strains (11/20) (Figures 4B,D).

The average number of streptococcal virulence genes expressed in all sequenced *emm*1.0 and *emm*12.0 strains was 125. The distribution of virulence genes varied among the strains, but was conserved and unique according to *emm* type (Figures 4B,D). Clades without *speH* and *speI* expression were found in *emm*12.0 strains (Figures 4C,D, highlighted in yellow). Genomic similarity analysis showed that *sAgs* were more conserved in *emm*1.0 strains than in the others. The frequency of *sda* expression in *emm*12.0 strains (20/22) was significantly higher than that in *emm*1.0 strains (15/20). Two copies of the bacteriophage encoding hyaluronate lyases *hylP* were present in *emm*1.0 strains, whereas three copies of *hylP* were observed in the *emm*12.0 strains (Figures 4B,D). Streptococcal toxin-encoding prophages and macrolide and tetracycline resistance integrative conjugative elements (ICEs) were found in both *emm*1 or *emm*12 isolates from Shenzhen with the following positive rates: φHKU16.vir (38.1%), φHKU488.vir (16.7%), Φ5005.1 (45.2%), Φ5005.2 (45.2%), and Φ5005.3 (11.9%), ICE-*emm*12 (35.7%), and ICE-HKU397 (54.8%).

Phylogenetic analysis using 33,067 core-gene SNPs revealed that *emm*1.0 and *emm*12.0 strains were clustered independently and diverged without recombination (Supplementary Figure 1). Additionally, for *emm*1.0 and *emm*12.0 strains, no clear independent cluster correlating to patients with OSAS or scarlet fever was found. The genetic diversity of *emm*12.0 strains (2902 SNPs) was higher than that of *emm*1.0 strains (384 SNPs) (Figures 4A,C). This difference in genetic diversity was also confirmed by the finding that *emm*12.0 strains presented more allelic variants (i.e., 12 allelic variants) than *emm*1.0 strains (i.e., 8 allelic variants).

## Discussion

GAS is a gram-positive bacterial pathogen that causes a wide range of clinical diseases, and antibiotics are effective agents for treating GAS infections. Although no GAS strains resistant to β-lactam antibiotics have been found, strains that are highly resistant to macrolides, lincosamides and streptomycin B have been identified globally since 1990 (Gherardi et al., 2015). All strains analyzed in this study were susceptible to penicillin and chloramphenicol, and most were susceptible to levofloxacin. Therefore, penicillin can remain the first-line drug for treating GAS infections in pediatric patients in China. The use of chloramphenicol and levofloxacin in children is limited due to the side effects of these antibiotics on the hematopoietic system and bone and joint development, respectively. Due to the high rate and degree of resistance to macrolides observed for GAS, these antibiotics cannot be used to effectively treat children who are allergic to lactam antibiotics. The use of tetracycline is also limited by a high rate and level of resistance among GAS as well as the side effects of tetracycline on bone and teeth. In the present study, 98.5% of the isolated GAS strains were sensitive to levofloxacin, and this finding is consistent with the rate observed among GAS strains isolated from children in Shanghai, China, but significantly different from the rate observed among GAS strains isolated from Chinese adults (Shen et al., 2018). The reason for this difference may be related to the medication habits of Chinese people. Levofloxacin is not widely prescribed to children due to its side effects on bone and joint development.

The PCR and WGS results in the present study indicate that macrolide-resistant and tetracycline-resistant strains mainly expressed *ermB* and *tetM*, respectively, which is consistent with the findings of previous studies (Feng et al., 2010; Liang et al., 2012; Tsai et al., 2020). Unexpectedly, expression of *lmrP*, a broad-spectrum drug efflux gene, and expression of *pmrA*, which potentially confers resistance to fluoroquinolone through drug efflux, were identified in all 42 strains by WGS. No penicillin-resistant strains or mutations in *pbp2x* were found. Nonetheless, a relationship exists between *pbp2x* gene variations and MIC values (Hayes et al., 2020; Musser et al., 2020; Vannice et al., 2020). Our WGS and MIC results showed that the *vanB*+ *vanrG*+ *vanuG–* and *vanB*+ *vanrG*+ *vanuG*+ strains were susceptible to vancomycin. These results underscore the need to better understand the relationship between antibiotic resistance phenotypes and resistance genes. ICEs have been proposed to play major roles in the selection and expansion of *emm*12 scarlet-fever outbreak lineages in Hong Kong and mainland China where antimicrobial usage patterns are elevated, highlighting their importance for *S. pyogenes* population structure (Davies et al., 2015; Shen et al., 2018; You et al., 2018; Jespersen et al., 2020). However, the role of ICEs in driving the global population structure of *S. pyogenes* has not been fully explored (Jespersen et al., 2020). The majority of characterized *S. pyogenes* exotoxins are carried by prophages, i.e., bacteriophages integrated in the bacterial chromosome. These SAg toxins, termed streptococcal pyrogenic exotoxins (spe), streptococcal mitogenic exotoxin Z (*smeZ*) and streptococcal superantigen (*ssa*), are amongst the most potent known activators of T cells (Blake et al., 2019).

A previous study evaluated time-dependent changes in the *emm* type prevalence of *S. pyogenes* (Meisal et al., 2010). In the present study, 10 different *emm* types, including 7 subtypes, were identified. The most prevalent types and subtypes were *emm*12.0 and *emm12*, followed by the *emm*1.0 and *emm1* (Figure 1). The prevalence of these two *emm* types is known to change over time (Ma et al., 2009; Liang et al., 2012; Li et al., 2020; You et al., 2020; Yu et al., 2020).

Previous studies confirmed the relationship between diseases caused by GAS infection and *emm* types (González-Abad and Alonso Sanz, 2020), although some other studies did not support this relationship. In the present study, the distribution of 15 diseases among the *emm*12.0 and *emm*2.0 types differed significantly. The *emm*2.0 strains were isolated from patients with 15 different diseases, whereas the *emm*12.0 strains were isolated mainly from patients with three diseases. These results indicate that the *emm* types of GAS strains infecting children in Shenzhen from 2016 to 2018 were associated with specific diseases.

*S. pyogenes* can cause infection by crossing human mucosal membranes and skin barriers. Prophage exotoxins enhance colonization fitness in epidemic scarlet fever-causing *S. pyogenes* (Brouwer and Barnett, 2020). The M protein and SAgs play a crucial role in the pathogenesis of *S. pyogenes* infections, and a close relationship exists between *emm* type and *sAg* distribution (Imöhl et al., 2017). The results of this study revealed differences in the frequency of *sAg* expression among *emm* types. Indeed, five *sAg* profiles were identified among GAS strains carrying six or more *sAgs*. Several *sAg* profiles were observed for each *emm* type, but only one or two genes were predominant for each type. Profiles B and E were most common among *emm*1.0 and its subtypes, whereas profiles A, C, and D were most common among *emm*12.0 and its subtypes. Thus, *sAgs* and their combinations are closely related to *emm* types. In this study, only a few strains were isolated for some diseases. We believe this could be related to the pathogenicity of *S. pyogenes* or to a lack of attention by researchers. In the past 30 years, most investigators have focused only on the relationship between *S. pyogenes* and postpartum infections, while the relationship with neonatal infections and vaginitis in girls has not received sufficient attention. Recent research on *S. pyogenes* shows that the pathogenicity of *S. pyogenes* in the vulva and vaginal mucosa of girls has become a concern (Donders et al., 2021; Hu et al., 2021). Although the amount of relevant information in this study is limited, the results provide insight into the relationship between *emm* type and vulvitis and vaginitis. More comprehensive research is needed.

The pathogenicity of GAS is related to the various virulence factors it produces. Pathogenic GAS has evolved to generate a large number of virulence factors, which promote its adhesion to host cells and invasion of deep tissues, ultimately leading to disease. SpeB can promote the spread of bacteria and their products in host tissues by degrading the tissue structure and can also degrade proteins and antimicrobial peptide LL-37 in order to resist immunity (Walker et al., 2014). SpeF is the main cause of pulmonary vascular permeability, which is sufficient to cause acute respiratory distress syndrome under the conditions of toxic shock-like syndrome caused by *S. pyogenes* (Matsumoto et al., 1999). Among all virulence factors, SAg is of particular concern. SAgs are proteins synthesized by ribosomes that have a relatively low molecular mass (∼22–28 kDa) and contain classic signaling peptides that are cleaved after secretion to release the mature toxin. SAgs function by activating T cells and are among the most powerful T-cell activators identified to date. At present, at least 14 genetically distinct SAgs have been characterized, and many of them are encoded within lysogenic bacteriophage or putative bacteriophage elements (Blake et al., 2019). Therefore, different strains encode different repertoires typically consisting of 3–6 distinct SAgs (Blake et al., 2019). Some studies reported a strong correlation between SAgs and disease categories (Smoot et al., 2002; Türk Dağı et al., 2018), and additional research showed that the distribution of *sAgs* correlates with disease categories and differs considerably among *emm* types (Murakami et al., 2002; Gergova et al., 2019). However, in the present study, we found that the *sAg* distributions were closely correlated to *emm* types but not to disease categories. The *emm*12.0 and especially *emm*2.0 strains were significantly associated with disease categories. Therefore, we speculate that variation in the distribution of *sAgs* is mainly due to the *emm* type, rather than the disease category.

In the present study, we generated the genome sequences of 42 *S. pyogenes emm*1 and *emm*12 strains. Streptococcal toxin-encoding prophage φHKU16.vir, φHKU488.vir, Φ5005.1, Φ5005.2, and Φ5005.3 in addition to the macrolide- and tetracycline-resistant ICE-*emm*12 and ICE-HKU397 elements were found amongst the Shenzhen strains. These results confirm our previous findings (You et al., 2018) of φHKU.vir, ICE-*emm*12 and ICE-HKU397 elements amongst multi-clonal *emm*12 strains of mainland China. Sequencing of more strains from China in the future will be important to determine the evolutionary pathway and population structure of the predominant *emm*1 and *emm*12 *S. pyogenes* strains.

The virulence factor streptococcal DNase sda1 was previously shown to interfere with the entrapment of bacteria through neutrophil extracellular traps and Toll-like receptor 9 (TLR9) signaling. This factor impairs plasmacytoid dendritic cell recruitment by reducing interferon (IFN)-1 levels at the site of infection and destabilizes DNA via the host protein HMGB1 (high mobility group box 1), which may decrease IFN-1 levels at the site of infection (1 levels at the site of infection (Uchiyama et al., 2012; Keller et al., 2019). Our WGS data showed that *sda* was specifically expressed by *emm*12.0 strains. In both *emm*12.0 and *emm*1.0 strains, the expression of *sda* showed no correlation with disease. *hylP* increases the virulence of GAS via the digestion of hyaluronic acid capsules (Singh et al., 2014). Our WGS data showed that the *hylP* expression in both *emm*12.0 and *emm*1.0 strains was specifically correlated with *emm* genotype but not with disease. Two copies of *hylP* were mainly distributed in the *emm*1.0 strains, and three copies of *hylP* were mainly distributed in the *emm*12.0 strains (Figure 4). Our study showed that variations in *hylP* might play roles in epidemic cloning expansion, but this needs to be further investigated.

In the present study, *emm*12.0 and its subtypes were present in 58.8% (201/342) of strains, and *emm*1.0 and its subtypes were present in 30.7% (105/342) of strains, which suggests that *emm*1.0 and *emm*12.0 were widespread and causing diseases in Chinese children from 2016 to 2018. The population structure and genetic diversity of these *emm* types were characterized by sequence typing and WGS (Supplementary Figure 1). The results showed that *emm*1.0 and *emm*12.0 each contained a stable clone, suggesting they had genetically diverged without recombination. However, the *emm*12.0 strains showed higher genetic diversity than the *emm*1.0 strains, suggesting that longer circulation of the former led to several robust clades with > 700 bootstrap replicates (Supplementary Figure 1). These observations are consistent with their epidemiological history. Horizontal transmission of virulence genes can occur between *emm* types. This should be considered in future GAS surveillance studies.

## Conclusion

In conclusion, antimicrobial agents commonly used to treat GAS infections are highly active against clinical strains. However, increasing macrolide resistance warrants analysis of the epidemiological characteristics of these strains. The molecular epidemiology of GAS in China has shown many differences from earlier reports, based on the present study along with the above-mentioned reports evaluating the molecular profiles of GAS strains collected in different time periods and geographical regions. In the future, multicenter studies including various diseases are necessary to assess whether our findings are affected by temporal and geographical changes.

## Data Availability Statement

The datasets presented in this study can be found in online repositories. The names of the repository/repositories and accession number(s) can be found below: https://www.ncbi.nlm.nih.gov/bioproject/, PRJNA743366.

## Ethics Statement

The studies involving human participants were reviewed and approved by this study was approved by the Research Ethics Committee of the Shenzhen Children’s Hospital. Written informed consent to participate in this study was provided by the participants’ legal guardian/nex of kin.

## Author Contributions

YZ and YY contributed to conception, design, and administrative support. QM, RZ, and YC provided study materials and patients. DY, YL, QL, WW, LH, and YB contributed to the collection and assembly of data, data analysis, and interpretation. DY and YL contributed to the manuscript writing. All authors contributed the final approval of manuscript.

## Conflict of Interest

The authors declare that the research was conducted in the absence of any commercial or financial relationships that could be construed as a potential conflict of interest.

## Publisher’s Note

All claims expressed in this article are solely those of the authors and do not necessarily represent those of their affiliated organizations, or those of the publisher, the editors and the reviewers. Any product that may be evaluated in this article, or claim that may be made by its manufacturer, is not guaranteed or endorsed by the publisher.

## Footnotes

1
https://www2.cdc.gov/vaccines/biotech/strepblast.asp
