# Supplementary material for: Molecular Characteristics of Streptococcus pyogenes Isolated From Chinese Children With Different Diseases
Source: Front Microbiol. 2021 Dec 9;12:722225. doi: 10.3389/fmicb.2021.722225 (PMC8696671; doi:10.3389/fmicb.2021.722225)
Supplement: Supplementary file 1 [file Data_Sheet_1.PDF]

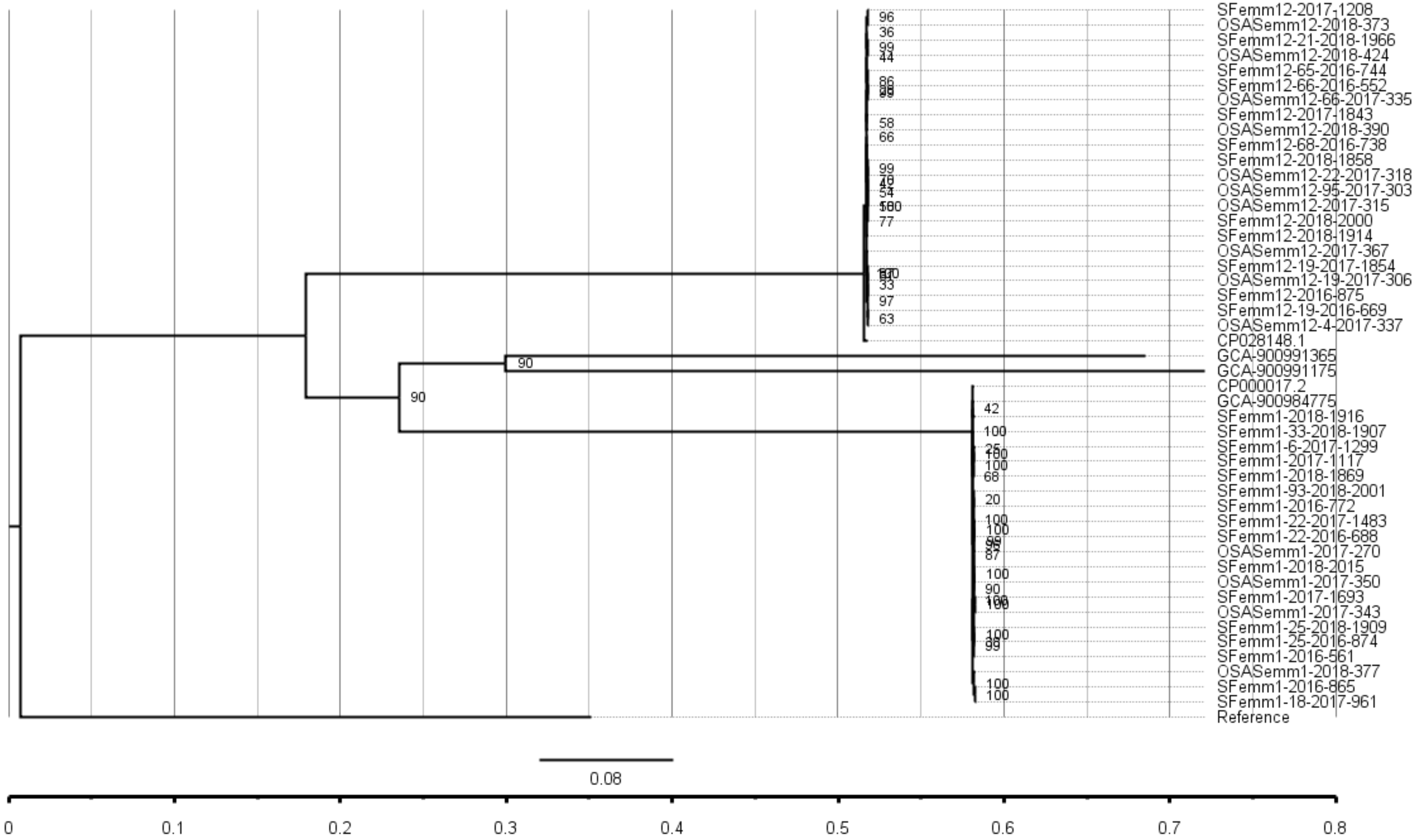

Supplementary Figure 1.

Figure S1. The phylogenetic relationship of selective *emm*1 and *emm*12 strains using whole genome core-gene SNPs. References indicates *S. pyogenes* strain JMUB1235 (AP017629). CP028148.1, GCA_900984775, GCA_900991175 and CP000017.2 are the assembly accessions of *S. pyogenes* strain TJ11-001, Bra048, NS678 and MGAS5005 respectively for references.
